# Predictive Performance Neutrophil-to-Lymphocyte Ratio of Acute Tonsillitis with Deep Neck Space Infection in Adult Patients

**DOI:** 10.1155/2023/8456427

**Published:** 2023-09-25

**Authors:** Sun Hwa Lee, Jong Seok Oh, Yun Hyung Choi, Ji Yeon Lim

**Affiliations:** ^1^Department of Emergency Medicine, Ewha Womans University Mokdong Medical Center, Ewha Womans University, 911-1 Mokdong, Yangcheon-gu, Seoul 07985, Republic of Korea; ^2^Seoul National University, Economics, 1 Gwanak-ro, Gwanak-gu, Seoul, Republic of Korea; ^3^Department of Emergency Medicine, Ewha Womans University Seoul Medical Center, Ewha Womans University, 260, Gonghang-daero, Gangseo-gu, Seoul 07804, Republic of Korea

## Abstract

The aim of this study was to examine the neutrophil-to-lymphocyte ratio (NLR) in patients diagnosed with a deep neck infection (DNI) to identify helpful indicators for the initial differential diagnosis. This study was conducted as a single-center, retrospective cohort study that utilized data from the electronic medical records of patients who visited the emergency department in a tertiary university hospital between February 2018 and April 2022. The study enrolled patients aged ≥18 years who were diagnosed with tonsillitis with or without DNI during the study period. The NLR of patients without DNI was 6.1 ± 5.03, and the NLR of patients with acute tonsillitis with DNI was 8.0 ± 5.67, showing significant differences. The rate of admission in the general wards (GWs) and ICUs was significantly higher in patients with DNI, and the length of hospital stay was also significantly longer in patients with DNI. Older age, male, lower body temperature, C-reactive protein, and NLR were significant independent risk factors for DNI in patients with tonsillitis. The cutoff value for predicting DNI in patients with body temperature <37.5 was 3.09. The NLR of patients with tonsillitis, especially those with normal body temperature, can be used to predict their prognosis.

## 1. Introduction

Tonsillitis accounts for a large percentage of patients visiting the emergency department (ED) for a sore throat [1]. Acute infection of the tonsil may involve the skin and mucous membranes and spread to surrounding connective tissues, forming an abscess, which could develop into deep neck infection (DNI) [2].

DNI is an infection of the head and neck that causes inflammation in the neck space or forms an abscess directly or through blood or lymphatic vessels. An antibiotic therapy leads to a decrease in the frequency, morbidity, and mortality in patients with DNI; however, some patients have a poor prognosis due to inappropriate use and overuse of antibiotics, including infection with antibiotic-resistant bacteria [3, 4].

Distinguishing upper respiratory infections, including tonsilitis, and DNI based only on the initial symptoms is often difficult, especially in patients who visit the ED for fever and sore throat. Failure to provide active treatment because DNI was overlooked as an upper respiratory infection will result in emergency intubation or tracheostomy because of abscess expansion. In addition, extensive incision and drainage may be required in the future, and the prognosis may be poor [5, 6]. Therefore, an accurate diagnosis of DNI in the ED is critical.

In 2001, Zahorec stated that the lymphocyte-to-neutrophil ratio (NLR) is a stress factor associated with systemic inflammation and a parameter that can predict the prognosis of patients with critical illness [7]. Since then, many studies have examined the relationship between NLR and various diseases [8–13].

The correlation between DNI and NLR in children was reported [14] previously, but it is not investigated in adult patients. Therefore, this study aimed to examine the NLR in patients diagnosed with DNI to identify helpful indicators for the initial differential diagnosis.

## 2. Methods

### 2.1. Study Design and Population

This study was conducted as a single-center, retrospective cohort study that utilized data from the electronic medical records (EMRs) of patients who visited the ED in a tertiary university hospital between February 2018 and April 2022. The study was approved by the institutional review board, and the need for informed patient consent was waived.

The study enrolled patients aged ≥18 years who were diagnosed with tonsillitis with or without DNI during the study period. Tonsillitis was diagnosed clinically in the presence of a sore throat or odynophagia and tonsil enlargement with or without exudates. By consultation with an otorhinolaryngologist, DNI was diagnosed if abscesses are present, such as ring enhancement in imaging examination such as computed tomography (CT), in patients with a history of tonsillitis or in whom pus was aspirated.

The exclusion criteria were as follows: (1) patients with an abscess history caused by foreign substances; (2) patients diagnosed with head, oral, and neck diseases such as cancer; (3) patients with immunocompromised status; (4) without a laboratory blood test; and (5) age <18 years.

### 2.2. Data Collection

One board-certified emergency physician collected data from the EMRs of coded cases without the knowledge of the aim of this study. The following variables were analyzed: demographics (sex and age), initial vital signs in the ED (systolic blood pressure, diastolic blood pressure, pulse rate, and body temperature), laboratory findings (white blood cell, hemoglobin, hematocrit, lymphocyte, neutrophil, platelet, C-reactive protein, blood urea nitrogen, creatinine, aspartate aminotransferase (AST), alanine aminotransferase (ALT), sodium, and potassium), ED treatment results (discharge, general ward (GW) admission, and intensive care unit (ICU) admission), and length of hospital stay.

### 2.3. Statistics

Categorical variables were analyzed using Pearson's chi-square test. Continuous variables were analyzed using the independent-samples *t*-test or Mann–Whitney *U* test, and they are presented as means with standard deviations and ranges. Logistic regression analysis was used to assess the association between variables and incidence of DNI.

IBM SPSS for Windows version 26.0 (IBM Corp., Armonk, NY, USA) was used. The area under the receiver operating characteristic (AUROC) curve analysis was performed with the DeLong method by using STATA Software version 17 (StataCorp., LLC, Texas, USA). A *p* value <0.05 was considered significant.

## 3. Results

### 3.1. Demographic, Laboratory, and Clinical Data

The EMRs of 432 patients were reviewed and analyzed for this study. In total, 309 patients were diagnosed with acute tonsillitis without DNI and 123 patients were diagnosed with acute tonsillitis with DNI.

Table 1 shows the result of the demographics and characteristics of patients.

The average age of the group without DNI was 40.2 ± 15.95 years, and the average age of the group with DNI was 46.8 ± 16.61 years, which was significantly different. A significant difference was found in the distribution of male patients between the two groups (50.8% vs. 73.2%). Diastolic blood pressure, body temperature, white blood cell, neutrophil, and lymphocyte counts showed significant differences.

The NLR of patients without DNI was 6.1 ± 5.03, and the NLR of patients with acute tonsillitis with DNI was 8.0 ± 5.67, showing significant differences. The rate of admission in the general wards (GWs) and ICUs was significantly higher in patients with DNI, and the length of hospital stay was also significantly longer in patients with DNI.

### 3.2. Risk Factors Associated with DNI

Table 2 shows the result of the univariate and multivariate regression analyses. In the univariate logistic regression analysis, age, male sex, systolic blood pressure, diastolic blood pressure, body temperature, white blood cell count, neutrophil count, lymphocyte count, C-reactive protein level, and NLR were significantly different among patients with and without DNI. Older age (odds ratio (OR) = 1.020; 95% confidence interval (CI) 1.003–1.038; *p* = 0.018), male sex (OR = 2.169; 95% CI 1.272–3.698; *p* = 0.004), lower body temperature (OR = 0.506; 95% CI 0.68–0.696; *p*  <  0.001), C-reactive protein (OR = 1.071; 95% CI 1.023–1.122; *p* = 0.003), and NLR (OR = 1.082; 95% CI 1.024–1.145; *p* = 0.005) were significant independent risk factors for DNI in patients with tonsillitis.

**Figure 1:**
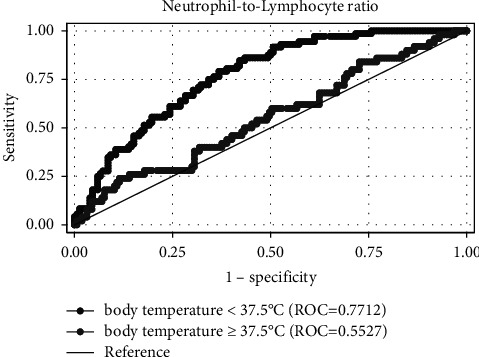
The ROC curve of neutrophil-to-lymphocyte ratio of patients with body temperature under 37.5°C and over 37.5°C.

The AUROC curve values for the NLR of patients with body temperature <37.5°C and ≥37.5°C for predicting DNI were 0.771 and 0.553, respectively (Figure 1). Based on the maximum sum of sensitivity and specificity, the cutoff value for predicting DNI in patients with body temperature <37.5 was 3.09. The sensitivity and specificity of the cutoff value in patients with body temperature <37.5 were 87.7% and 57.0%, respectively.

### 3.3. Comparison between Patients with High and Low NLR

Based on the NLR cutoff of ≥3.09, patients were divided into the low NLR group and the high NLR ratio group (Table 3). No significant differences were noted in age and blood pressure between the two groups. The pulse rate and body temperature in the higher NLR group were significantly different from those in the lower NLR group. A significantly higher GW admission rate was associated with NLR ≥3.09 than with <3.09 (GW admission rate 7.0% vs. 28.1%, respectively (*p*  <  0.001)). The average hospital length between each group was 0.4 and 1.8 days, showing significant differences. This shows that NLR levels are suitable for predicting the prognosis of patients, and if those levels exceed 3.09, the hospital length is extended and the prognosis is poor.

## 4. Discussion

In this study, we found that NLR can be one of the predictors of the likelihood of tonsillitis progressing into DNI. Specifically, NLR is a more useful factor in patients with tonsillitis and normal body temperature.

If the inflammation spread because of inaccurate diagnoses due to the inability to distinguish between DNI and simple tonsillitis, mediastinitis, pericarditis, pneumonia, meningitis, and sepsis may occur [15–17].

Tonsillitis is less likely to result in DNI in adult patients than in children [18]. Therefore, when diagnosing adult patients with tonsillitis who visited the ED, there are cases where the complication is overlooked. However, in patients with DNI, upper respiratory obstruction may occur, and airway intubation or tracheostomy may be required to preserve the airway. Adult patients with DNI experience pain in the invasion area, fever, and malaise. Drooling and hot photo voice may develop [19]. The NLR value was higher in tonsillitis patients with DNI than in those without DNI.

NLR can be calculated in a basic blood laboratory test, and it is a marker that can be easily, conveniently, and inexpensively checked in the ED. de Jager et al. demonstrated the association between community-acquired pneumonia and NLR [20], and Lee et al. studied that NLR could be a predictor of prognosis in cellulitis [21]. In addition, the usefulness of NLR as a prognostic factor for cardiovascular disease and cancer has been studied, and a study analyzed the relationship between mortality and NLR in older patients [11, 22]. Recently, many studies on the role of NLR in coronavirus disease-2019 have also been published [23–25].

Among patients with tonsillitis, the GW and ICU admission rates were significantly higher in those with DNI than in those without DNI, and the hospital length was also significantly longer. Therefore, making an accurate diagnosis of DNI in primary care in the ED is important. In this study, the OR of NLR is higher than that of C-reactive protein, a common indicator of severity and prognosis in infection diseases.

Forger reported that the normal range of the NRL is between 0.78 and 3.53. In the present study [26], the cutoff NLR that could predict DNI development was 3.09. According to the cutoff criteria, patients were divided into the low NLR group and the high NLR group. The GW admission rate in the high NRL group was higher than that in the low NLR group (28.1%, 7.0% *p* value <0.001), and the average hospital length between each group was 0.4 and 1.8 days, showing significant differences. Thus, NLR levels are suitable for predicting the prognosis, and if those levels exceed 3.09, the hospital length is prolonged and the prognosis is poor.

In Baglam's study, an NLR cutoff of 5.4 was set to predict the risk of DNI in pediatric patients, with a sensitivity of 96% and a specificity of 83% (85% positive predictive value and 95.4% negative predictive value) [14]. Fiorella et al. reported that a cutoff of 17.5 was set to determine the risk of cervical necrotizing fasciitis, with a sensitivity and specificity of 50% and 84.9%, respectively, and a cutoff of 8.2 to predict systemic septic involvement, with a sensitivity and specificity of 74.2% and 61.5% [27]. In a study of DNIS due to periodontal infection, the optimal cutoff value for NLR for length of stay ≥2 days was 4.65 [28]. Although the cutoff value varies between studies and the sensitivity and specificity are not satisfactory, we believe that this suggests the need for further studies.

A strength of this study is that the NLR of patients with tonsillitis, especially those with a normal body temperature, can be used to predict their prognosis. Thus, if CT is performed to determine DNI, then the NLR should be considered concurrently. If the ratio is ≥3.09, even with normal body temperature, more aggressive, prompt, and timely treatment is required to prevent a poor prognosis.

This study has limitations in the interpretation of the findings. First, the study only included patients from one general hospital retrospectively. Additional studies with multicenter, prospective designs are warranted. Second, blood tests were not investigated in all patients. Third, tonsillitis is often diagnosed clinically, and treatment was provided without additional examinations. Finally, because tonsillitis is diagnosed clinically, the diagnostic criteria may vary among physicians.

In patients with tonsillitis and high NLR, further evaluation including imaging such as CT should be performed to determine the extension of DNI. Even with normal body temperature, if NLR ≥3.09, more aggressive and prompt treatment is required.

## 5. Conclusions

The NLR of patients with tonsillitis, especially those with normal body temperature, can be used to predict their prognosis. Thus, if CT is performed to determine DNI, then the NLR should be considered concurrently. If the ratio is ≥3.09, even with normal body temperature, more aggressive, prompt, and timely treatment is required to prevent a poor prognosis.

## Figures and Tables

**Table 1:** Demographics and characteristics of patients.

Variables	Acute tonsillitis without DNI	Acute tonsillitis with DNI	*p* value
	*n* = 309	*n* = 123	
Age	40.2 ± 15.95	46.8 ± 16.61	**<0.001**
Male sex	157 (50.8%)	90 (73.2%)	**<0.001**
Systolic blood pressure (mmHg)	131.3 ± 17.88	136.8 ± 19.66	0.008
Diastolic blood pressure (mmHg)	78.4 ± 11.75	82.1 ± 13.23	**0.004**
Pulse rate (beats/min)	94.1 ± 17.57	92.4 ± 18.21	0.390
Body temperature (°C)	37.6 ± 0.98	37.3 ± 0.74	**0.006**
Laboratory test			
Hemoglobin (g/dL)	13.8 ± 1.50	14.1 ± 1.39	0.087
Hematocrit (%)	39.8 ± 4.07	40.5 ± 3.80	0.073
White blood cell (10^9^/L)	11.3 ± 4.59	13.7 ± 3.64	**<0.001**
Neutrophil (%)	71.2 ± 13.93	77.6 ± 7.89	**<0.001**
Lymphocyte (%)	19.1 ± 12.43	13.6 ± 7.12	**<0.001**
ANC (×10^3^/*μ*L)	8.4 ± 4.45	10.8 ± 3.52	**<0.001**
Platelet (10^9^/L)	236.0 ± 66.96	247.2 ± 65.15	0.113
Blood urea nitrogen (mg/dL)	12.3 ± 5.20	14.07 ± 6.41	**0.003**
Creatinine (mg/dL)	0.8 ± 0.37	0.9 ± 0.26	0.320
C-reactive protein (mg/dL)	4.7 ± 5.32	7.9 ± 7.39	**<0.001**
Aspartate aminotransferase (IU/L)	26.9 ± 18.88	25.3 ± 19.6	0.427
Alanine aminotransferase (IU/L)	289.0 ± 37.52	30.7 ± 35.60	0.661
Sodium (mmol/L)	137.3 ± 2.84	136.9 ± 2.79	0.179
Potassium (mmol/L)	3.9 ± 0.36	3.9 ± 0.36	0.059
NLR	6.1 ± 5.03	8.0 ± 5.67	**0.003**
GW admission	25 (8.1%)	72 (58.5%)	**<0.001**
ICU admission	2 (0.6%)	5 (4.1%)	**0.022**
Hospital length	0.4 ± 1.73	4.0 ± 8.86	**<0.001**

DNI, deep neck infection; NLR, neutrophil-lymphocyte ratio; GW admission, general wards admission; ICU admission, intensive care unit admission. Bold values are statistically significant with a *p* value less than 0.05.

**Table 2:** Logistic regression analysis of tonsillitis with DNI or without DNI.

Variables	Univariate analysis			Multivariate analysis		
	OR	95% CI	*p* value	OR	95% CI	*p* value
Age	1.024	1.011–1.037	**<0.001**	1.020	1.003–1.038	**0.018**
Male sex	2.640	1.672–4.170	**<0.001**	2.169	1.272–3.698	**0.004**
Systolic blood pressure (mmHg)	1.017	1.005–1.029	**0.006**	1.004	0.986–1.021	0.696
Diastolic blood pressure (mmHg)	1.027	1.008–1.046	**0.004**	1.026	1.000–1.054	0.051
Pulse rate (beats/min)	0.995	0.983–1.007	0.387			
Body temperature (°C)	0.734	0.578–0.931	**0.011**	0.506	0.368–0.696	**<0.001**
Laboratory test						
Hemoglobin (g/dL)	1.137	0.981–1.317	0.087			
Hematocrit (%)	1.049	0994−1.108	0.082			
White blood cell (10^9^/L)	1.130	1.076–1.187	**<0.001**			
Neutrophil (%)	1.049	1.027–1.071	**<0.001**			
Lymphocyte (%)	0.944	0.920–0.968	**<0.001**			
Platelet (10^9^/L)	1.002	0.999–1.006	0.117			
Blood urea nitrogen (mg/dL)	1.055	1.017–1.094	**0.004**	0.991	0.943–1.042	0.728
Creatinine (mg/dL)	1.238	0.713–2.308	0.406			
C-reactive protein (mg/dL)	1.081	1.044–1.119	**<0.001**	1.071	1.023–1.122	**0.003**
Aspartate aminotransferase (IU/L)	0.995	0.983–1.007	0.421			
Alanine aminotransferase (IU/L)	1.001	0.996–1.007	0.668			
Sodium (mmol/L)	0.951	0.883–1.024	0.180			
Potassium (mmol/L)	1.757	0.977–3.161	0.060			
NLR	1.068	1.028–1.110	**0.001**	1.082	1.024–1.145	**0.005**

DNI, deep neck infection; OR, odd ratio; 95% CI, 95% confidence interval; NLR, neutrophil-lymphocyte ratio; GW admission, general wards admission; ICU admission, intensive care unit admission. Data in parentheses are 95% confidence intervals, conducted on variables with a *p* value of <0.05 on univariate analysis. Bold values are statistically significant with a *p* value less than 0.05.

**Table 3:** Comparison between the patients with higher and lower NLR group.

Variables	Low NLR group <3.09	High NLR group ≥3.09	*p*-value
	*n* = 115	*n* = 317	
Age	42.6 ± 17.00	41.9 ± 16.19	0.724
Male sex	54 (47.0%)	193 (60.9%)	**0.010**
Systolic blood pressure (mmHg)	134.6 ± 21.69	132.28 ± 17.26	0.260
Diastolic blood pressure (mmHg)	81.7 ± 13.89	78.6 ± 13.90	0.210
Pulse rate (beats/min)	85.4 ± 19.89	69.6 ± 17.14	**<0.001**
Body temperature (°C)	36.9 ± 0.64	37.7 ± 0.9131	**<0.001**
Laboratory test			
Hemoglobin (g/dL)	13.7 ± 1.51	14.0 ± 1.46	0.054
Hematocrit (%)	39.5 ± 4.20	40.2 ± 3.92	0.108
White blood cell (10^9^/L)	8.2 ± 3.30	13.3 ± 4.06	**<0.001**
Neutrophil (%)	56.1 ± 11.0	79.2 ± 6.61	**<0.001**
Lymphocyte (%)	33.0 ± 10.5	12.1 ± 4.83	**<0.001**
Platelet (10^9^/L)	241.5 ± 69.94	238.3 ± 65.42	0.668
Blood urea nitrogen (mg/dL)	14.0 ± 5.89	12.4 ± 5.47	**0.008**
Creatinine (mg/dL)	0.8 ± 0.17	0.9 ± 0.38	0.051
C-reactive protein (mg/dL)	1.8 ± 2.42	7.0 ± 6.48	**<0.001**
Aspartate aminotransferase (IU/L)	28.8 ± 18.68	25.6 ± 19.17	0.134
Alanine aminotransferase (IU/L)	33.1 ± 43.90	28.2 ± 34.02	0.225
Sodium (mmol/L)	138.3 ± 2.57	136.7 ± 2.81	**<0.001**
Potassium (mmol/L)	3.9 ± 0.36	3.9 ± 0.36	0.431
NLR	1.9 ± 0.69	8.3 ± 5.18	**<0.001**
With DNI	15 (13.0%)	108 (34.1%)	**<0.001**
GW admission	8 (7.0%)	89 (28.1%)	**<0.001**
ICU admission	1 (0.9%)	6 (1.9%)	0.457
Hospital length	0.4 ± 2.29	1.8 ± 5.87	**0.017**

NLR, neutrophil-lymphocyte ratio; DNI, deep neck infection; GW admission, general wards admission; ICU admission, intensive care unit admission. Bold values are statistically significant with a *p* value less than 0.05.

## Data Availability

The data used to support the findings of this study are available from the corresponding author upon request.

## References

[1] Gilley D. R., Virdi G. S., Namin A. W., Dooley L. M. (2021). Utility of CT in the workup of adults with sore throat in the emergency department. *The American Journal of Emergency Medicine*.

[2] Perina V., Szaraz D., Harazim H., Urik M., Klabusayova E. (2022). Paediatric deep neck infection—the risk of needing intensive care. *Children*.

[3] Velhonoja J., Lääveri M., Soukka T., Irjala H., Kinnunen I. (2020). Deep neck space infections: an upward trend and changing characteristics. *European Archives of Oto-Rhino-Laryngology*.

[4] Sethi D. S., Stanley R. E. (1994). Deep neck abscesses--changing trends. *Journal of Laryngology & Otology*.

[5] Lin Y., Gao W., Yue H. (2021). A novel risk score for the prediction of airway management in patients with deep neck space abscess: a multicenter retrospective cohort study. *Journal of Intensive Care*.

[6] Boscolo-Rizzo P., Stellin M., Muzzi E. (2012). Deep neck infections: a study of 365 cases highlighting recommendations for management and treatment. *European Archives of Oto-Rhino-Laryngology*.

[7] Zahorec R. (2001). Ratio of neutrophil to lymphocyte counts-rapid and simple parameter of systemic inflammation and stress in critically ill. *Bratislavske Lekarske Listy*.

[8] Diem S., Schmid S., Krapf M. (2017). Neutrophil-to-Lymphocyte ratio (NLR) and Platelet-to-Lymphocyte ratio (PLR) as prognostic markers in patients with non-small cell lung cancer (NSCLC) treated with nivolumab. *Lung Cancer*.

[9] Cedres S., Torrejon D., Martinez A. (2012). Neutrophil to lymphocyte ratio (NLR) as an indicator of poor prognosis in stage IV non-small cell lung cancer. *Clinical and Translational Oncology*.

[10] Feng J.-F., Huang Y., Chen Q.-X. (2014). Preoperative platelet lymphocyte ratio (PLR) is superior to neutrophil lymphocyte ratio (NLR) as a predictive factor in patients with esophageal squamous cell carcinoma. *World Journal of Surgical Oncology*.

[11] Afari M. E., Bhat T. (2016). Neutrophil to lymphocyte ratio (NLR) and cardiovascular diseases: an update. *Expert Review of Cardiovascular Therapy*.

[12] Duchesne J. C., Tatum D., Jones G. (2017). Multi-institutional analysis of neutrophil-to-lymphocyte ratio (NLR) in patients with severe hemorrhage: a new mortality predictor value. *Journal of Trauma and Acute Care Surgery*.

[13] Feghali J., Kim J., Gami A., Rapaport S., Caplan J. M., McDougall C. G. (2021). Monocyte-based inflammatory indices predict outcomes following aneurysmal subarachnoid hemorrhage. *Neurosurgical Review*.

[14] Baglam T., Binnetoglu A., Yumusakhuylu A. C., Gerin F., Demir B., Sari M. (2015). Predictive value of the neutrophil-to-lymphocyte ratio in patients with deep neck space infection secondary to acute bacterial tonsillitis. *International Journal of Pediatric Otorhinolaryngology*.

[15] Koç A. K., Alakhras W. M., Acıpayam H., Koçak H. E., Kayhan F. T. (2016). Seven years of experience in 160 patients with deep neck infection. *Imaging (MRI)*.

[16] Wahab D., Bichard J., Shah A., Mann B. (2013). Just a sore throat? Uncommon causes of significant respiratory disease. *Case Reports*.

[17] Zilberstein B., Cleva R. D., Testa R. S., Sene U., Eshkenazy R., Gama-Rodrigues J. J. (2005). Cervical necrotizing fasciitis due to bacterial tonsillitis. *Clinics*.

[18] Walls R. M., Hockberger R., Gausche-Hill M., Erickson T. B., Wilcox S. (2022). *Rosen’s Emergency Medicine, Concepts and Clinical Practice*.

[19] Brahim J. S., Ord R. A. (2017). Severe head and neck infections. *Endodontic Microbiology*.

[20] de Jager C. P., Wever P. C., Gemen E. F. (2012). The neutrophil-lymphocyte count ratio in patients with community-acquired pneumonia. *PLoS One*.

[21] Lee K. H., Lee S. Y., Oh C. H., Park K. (2022). Role of the neutrophil-to-lymphocyte ratio as a predictor of prognosis in cellulitis. *Korean Journal of Dermatology*.

[22] Angkananard T., Anothaisintawee T., McEvoy M., Attia J., Thakkinstian A. (2018). Neutrophil lymphocyte ratio and cardiovascular disease risk: a systematic review and meta-analysis. *BioMed Research International*.

[23] Yang A.-P., Liu J. P., Tao W. Q., Li H. M. (2020). The diagnostic and predictive role of NLR, d-NLR and PLR in COVID-19 patients. *International Immunopharmacology*.

[24] Citu C., Gorun F., Motoc A. (2022). The predictive role of NLR, d-NLR, MLR, and SIRI in COVID-19 mortality. *Diagnostics*.

[25] Kerboua K. E. N. L. R. (2021). NLR: a cost-effective nomogram to guide therapeutic interventions in COVID-19. *Immunological Investigations*.

[26] Forget P., Khalifa C., Defour J.-P., Latinne D., Van Pel M.-C., De Kock M. (2017). What is the normal value of the neutrophil-to-lymphocyte ratio?. *BMC Research Notes*.

[27] Fiorella M. L., Greco P., Madami L. M., Giannico O. V., Pontillo V., Quaranta N. (2020). New laboratory predictive tools in deep neck space infections. *Acta Otorhinolaryngologica Italica*.

[28] Gallagher N., Collyer J., Bowe C. M. (2021). Neutrophil to lymphocyte ratio as a prognostic marker of deep neck space infections secondary to odontogenic infection. *British Journal of Oral and Maxillofacial Surgery*.

